# Dominant vs. non-dominant hip comparison in bone mineral density in young sporting athletes

**DOI:** 10.1007/s11657-019-0605-2

**Published:** 2019-05-25

**Authors:** James A. van Santen, Claudio Pereira, Maria T. Sanchez-Santos, Cyrus Cooper, Nigel K. Arden

**Affiliations:** 1 Oxford NIHR Musculoskeletal Biomedical Research Unit, Nuffield Department of Orthopaedics, Rheumatology and Musculoskeletal Sciences, University of Oxford, Oxford, UK; 2 Arthritis Research UK Centre for Sport, Exercise and Osteoarthritis, Nuffield Department of Orthopaedics, Rheumatology and Musculoskeletal Sciences, University of Oxford, Oxford, UK; 3 Nuffield Department of Orthopaedics, Rheumatology and Musculoskeletal Sciences, Botnar Research Centre, University of Oxford, Old Road, Oxford, OX3 7LD UK; 4 MRC Lifecourse Epidemiology Unit, Southampton General Hospital, University of Southampton, Southampton, UK

**Keywords:** Bone density, DXA, Sport, Hip, Dominant foot

## Abstract

***Summary*:**

To explore differences in bone mineral density (BMD) between dominant and non-dominant hip within levels of sport impact. BMD was higher in the non-dominant hip in high-impact sports, whereas the dominant hip had increased BMD for low-impact sports. The side-to-side differences were relatively small and not clinically relevant.

**Purpose:**

It is unknown whether there is difference in BMD at the hip between dominant and non-dominant sides in young athletes. The aims of this study were to explore the dominant–non-dominant differences in hip BMD in young athletes participating in low- and high-impact sports and to assess the effect of ground force impact on BMD.

**Methods:**

Data was collected on University of Oxford athletes and controls (CG) between 2016 and 2018. Athletes were classified into two groups: high-impact sports (HIG) and low-impact sports (LIG). Total and regional measurements of both hips’ BMD were recorded using a dual-energy X-ray absorptiometry (DXA). Linear regression method was used to assess differences in BMD between and within groups.

**Results:**

One hundred ninety-four athletes (HIG: *n* = 89, LIG: *n* = 105) and 48 controls were included in this study. Total hip and femoral neck BMD was higher in athletes compared to the CG (*p* < 0.01), with HIG recording highest levels of BMD. The BMD difference between the dominant and non-dominant sides was significant in the LIG, with BMD being higher in the dominant side. Conversly, BMD was higher in the non-dominant hip within the HIG. However, the hip asymmetries were not clinically relevant (%BMD difference < 3%). A significant interaction between side and sport group on BMD was observed.

**Conclusions:**

High-impact sports had significantly higher BMD compared with low-impact sports and CG. BMD in the dominant hip was significantly higher for the LIG and lower in the HIG; however, differences were not clinically relevant.

**Electronic supplementary material:**

The online version of this article (10.1007/s11657-019-0605-2) contains supplementary material, which is available to authorized users.

## Introduction

Bone mineral density (BMD) is an important indicator of skeletal health, integrity, and strength [1]. Dual-energy X-ray absorptiometry (DXA) has been regarded for many decades as the gold standard for determining the true BMD and bone mineral content (BMC) of in vivo subjects due to its high precision and reproducibility [2]. As the DXA scan also provides a detailed breakdown of body composition, including fat mass and lean mass, the use of DXA is increasingly being used to characterise BMD and body composition of athletes in a sporting environment [2, 3].

The International Society for Clinical Densitometry’s guidelines (2015) recommends measuring BMD at both the anteroposterior spine and either hip to assess bone health [4]. Unilateral measurements of proximal femoral BMD are routinely performed to minimise the scan time, medical costs, and the patient’s exposure to ionising radiation [5, 6]. In clinical practice, the measurement of the non-dominant hip is preferred based on the assumption that the non-dominant side is less physically active and therefore exposed to less stress and impact, resulting in lower BMD. Determining the non-dominant hip has conventionally been identified using the contralateral side to the forearm dominance [7], resulting in the scanning of the left hip in 90% of the time. This makes the assumption that ambidexterity or cross-dominance does not exist in humans, potentially leading to erroneous selection of hip with the lowest BMD. One main issue with most scientifically published hip BMD studies is that they do not specify if dominance was defined by footedness or handedness/forearm dominance.

Several studies have suggested that there are no significant differences between the right and left hips, reporting high correlations between hips (*r* > 0.9) [8–11], and that leg dominance does not have an influence BMD [12, 13]. However, other studies have found an effect on BMD caused by dominance [5, 14–16]. In a large study of 2372 postmenopausal women (mean age 56.6 years), Petley and colleagues [6] found statistically significant differences between the right and left proximal femurs in the femoral neck; however, these differences were relatively small and not clinically relevant. The authors concluded that only a small benefit would be gained from performing bilateral femoral neck BMD measurements.

Participation in sport and physical activity has been shown to have positive effects on BMD and bone adaptation responses [17–19]. Research has shown that BMD in athletes is significantly greater than sedentary controls and that it is highest in athletes who participate in high-impact exercise, defined as activities involving running, jumping, and weight lifting [20–24]. This may be due to the types of movements in sports, such as running, producing repetitive loading consisting of moderately high ground reaction forces of 2–3 times body weight [25, 26]. Conversely, low-impact sports, such as cycling and swimming, have been shown to have little or negative effects on long-term BMD [27], due to the non-weight-bearing and low-impact aspects of the sports. Greenway and colleagues [28] found that there was no difference in BMD between swimmers and control during a 5-year follow-up period and concluded that long-term swim training participation did not compromise regional BMD. BMD can also be indirectly affected by repeated muscle contractions with repetitive, vigorous movements which increase the mechanical loading and strain on the skeleton, which would lead to positive changes in BMD [29]. Lee and Kim [29] stated that BMD measurements in the athletic population need to consider characteristics of the sport type and the bone-loaded regions.

Since a direct association between physical activity/load stress and BMD is accepted [17–19, 22], it is expected that once physical activity/load stress is increased, that BMD is also increased. As load stress is never homogenous between dominant and non-dominant sides, the enhanced loading patterns of the dominant foot in high-impact athletes may result in larger asymmetries between hip BMDs compared with low-impact athletes and non-athletes. Wu and Colleagues [30] found that the take-off hip had a 4–9% increase in BMD compared with the landing hip in female gymnasts, this difference being attributed to increased ground reaction forces during take-off than landing. This suggests that the unilateral loading of the dominant hip has an intensive loading impact effect on the skeleton and enhances the development of bone. This unilateral loading effect was observed in ten-pin bowlers with the loaded side having increased bone area and BMD compared with the non-loaded slide leg [31]. Chikibeck and colleagues [32] found that an older female population (age = 57.4) recorded higher BMD percentage differences favouring the dominant side compared with a younger (age = 20.9) group (5.2% vs. 1.9%, respectively), implying that the greater use of dominant limbs in everyday activities across a longer lifespan resulted in an increased stimulation of bone on the dominant side.

Most previous studies have focused their research on side asymmetries in patients at risk of presenting osteoporosis and/or comparing population in terms of BMD; however, to the best of our knowledge, none has studied hip asymmetries in young athletes participating in low- and high-impact sports. Therefore, the aims of this study were to assess differences in BMD at the hip between dominant and non-dominant sides in young athletes participating in low- and high-impact sports, and in a non-athletic group, and to explore the effect of ground force impact on hip BMD between impact groups.

## Methods

### Participants

A total of 301 participants (*n* = 150 males, *n* = 151 females), mean age of 24.4 ± 4.7 years old, from the University of Oxford were recruited. Sporting participants were recruited through university sports teams via the team management or the sports physiologist, whilst the control group was recruited through advertisement in college newsletters and recruitment posters. All participants were scanned using a GE Lunar iDXA between May 2016 and June 2018. Participants were categorised into cases and control groups, with the case group (79%) being comprised of active university sporting club members. Cases were then divided into two groups according to the amount of ground force impact the sport has on their body (high-impact sports group (HIG) (*n* = 105) and low-impact sports group (LIG) (*n* = 134)). The HIG included sporting participants from rugby, powerlifting, and athletics clubs, where the body and hips are subjected to repeated high-ground impact forces and stresses during play. The LIG included rowing, cycling, and swimming clubs, where there is little to no ground force impact placed on the body during play and thus non-weight bearing. The control group (CG) (*n* = 62, 21%) included non-sporting students that were not part of a sporting team or participated in physical activity on a regular basis. Both athlete groups reported higher levels of physical activity and sport per week compared with the CG. The average hours of physical activity per week was 16.8 ± 6.4 h, 8.7 ± 3.3 h, and 1.8 ± 2.6 h per week in the LIG, HIG, and CG, respectively.

Nineteen participants identified as being foot ambidextrous, 40 participants who did not specify a dominant side, and one who did not agree to a DXA scan were excluded from the analysis.

### Ethics

A local NHS Research Ethics Committee (South Central—Oxford C Research Ethics Committee; ref: 16/SC/0187) provided ethical approval for the study and all required authorisations were attained prior to the start of the study. Participants received written and oral information regarding the study at least 24 hours prior to providing consent. Participant informed consent was acquired for all subjects in person, in writing, and on the same day when the DXA scan was performed.

### Procedures

Following the signing of consent forms, a questionnaire, based on health and lifestyle, was administered to all participants prior to the DXA scan. This included medical and family history, previous and current injuries, and physical activity as well as past and present lifestyle practices including smoking, alcohol, and dairy consumption. Preference of footedness (e.g. foot they preferred to kick a ball with) and handedness (e.g. writing side preference) were assessed within the questionnaire, and the dominant foot preference was used to determine the dominant and non-dominant sides.

BMD was measured using a DXA scanner at the Nuffield Orthopaedic Centre (University of Oxford, Oxford, UK). Total and regional measurements (femoral neck, Ward’s triangle area, trochanter, and shaft) of both hip’s BMD (g/cm^2^), *Z*-scores, BMC (g), and area (cm^2^) were recorded.

Quality assurance (QA) was performed before all scan sessions in accordance with the manufacturer’s guidelines to ensure that the scanner was calibrated and working appropriately. Throughout the recruitment phase (2 years), the scanner’s precision error varied between 0.25 and 0.27%, based on the QA’s results. All scans were performed by the same radiographer and conducted in accordance with the GE Healthcare Lunar’s Operator’s manual (Revision 9, March 2012, GE Medical Systems Lunar, WI, USA).

Participants were asked to wear light clothing or hospital gowns, and all metallic and plastic artefacts were removed prior to the scan. Height was measured by a SECA Leicester Height Measure to the nearest millimetre and total body mass was calculated from the DXA report to the nearest 0.1 kg. Body mass index (BMI) was calculated as the ratio of the weight to the square of height in metres (kg/m^2^). Participants were then positioned in a supine position on the scanning bed with the radiographer ensuring the correct body position (following the GE Healthcare Lunar’s Operator’s manual guidelines) for consistency across all scans. Both hips were scanned independently and sequentially. Image post-processing was performed by the operator immediately after the scan by identifying anatomical landmarks and manually defining regions of interest (ROI) within the acquired images using the DXA’s own software (Lunar enCORE, version 14.10.022, copyright 1998–2012, GE Medical Systems Lunar, WI, USA). The Lunar enCORE software then calculated regional densities and compositions. *Z*-scores were calculated based on the combined National Health and Nutrition Examination Survey (NHANES) (ages 20–30 years)/Lunar (ages 20–40 years) Femur Reference Population (v113) and matched for age, gender, and ethnicity. *T*-scores were not reported as the 2015 Official ISCD Positions [4] state that for BMD reporting in female prior to menopause and in males younger than age 50 years old *Z*-score are preferred.

### Statistical analysis

Statistical analysis was undertaken using Stata version 13.1 statistical software (StataCorp, College Station, TX). Participant’s characteristics and bone density measurements were summarised by the mean and the standard deviation (SD). Normal distribution of each outcome was assessed by visual inspection. Bivariate correlations between both hips were assessed using Pearson’s correlation test.

Standard linear regression analyses unadjusted and adjusted for potential confounders (age, sex, and BMI) were conducted to assess the association of impact group on BMD, *Z*-score, BMC, and area at total hip and femoral neck with the CG used as the reference group. Mean BMD of both sides was calculated for this analysis.

Differences in bone density between hips (dominant vs. non-dominant foot) on BMD, *Z*-score, BMC, and area were assessed using linear regression with generalised estimating equation (GEE) methods to account for hip correlation within the same individual. To assess whether hip asymmetries differed between sport groups, an interaction term side*sport group was added to the model. If a statistically significant interaction was found, we examined the estimates for the association between side and BMD stratified by sport group.

Percentage differences between sides were calculated using the following formula: Side BMD % difference = (((Dominant BMD − Non-dominant BMD)/Non-dominant BMD)*100). Clinically important difference was set at 3% or higher, based on the GE Healthcare Lunar’s Operator’s manual and the operator precision error (which also includes the scanner precision error) at the location where the exams where performed (Nuffield Orthopaedic Centre).

Statistical threshold of significance was defined as *p* value < 0.05.

## Results

### Descriptive data

Descriptive data for all 242 participants by sporting groups and CG are presented in Table 1. Ninety percent of all participants were right foot dominant and this distribution is similar between groups. Thirty-seven percent of participants were in the HIG and 43% in the LIG. The mean ± SD age of HIG (23.4 ± 4.1) and LIG (23.7 ± 3.9) was significantly lower than the CG (28.9 ± 5.6) at *p* < 0.001. LIG were significantly taller than participants classified in the HIG and CG, and both sporting groups were significantly heavier than the CG *p* < 0.001. Percent body fat was significantly higher in the CG with the LIG having the lowest levels of body fat.

**Table 1:** Descriptive data stratified by group

	All	High impact	Low impact	Control group
*n*	242	89	105	48
Age (years)	24.6 (4.9)	23.4 (4.1)	23.7 (3.9)	28.9 (5.6)
Sex, female *n* (%)	109 (45.0%)	37 (41.6%)	42 (40.0%)	30 (62.5%)
Height (cm)	176.0 (9.9)	174.2 (8.3)	180.1 (9.1)	170.5 (10.4)
Weight (kg)	72.6 (12.8)	74.0 (13.9)	74.8 (11.1)	65.4 (11.6)
BMI (kg/m^2^)	23.3 (2.8)	24.2 (3.3)	22.9 (1.9)	22.4 (2.8)
Lean mass (kg)	55.0 (12.8)	56.4 (12.4)	58.9 (11.3)	43.8 (9.7)
Total fat mass (%)	21.4 (8.6)	20.7 (7.5)	18.0 (6.8)	30.3 (8.0)
Right foot dominant (%)	90.5	86.5	91.4	95.8
Physical activity and sport (hours/week)	10.9 (7.5)	8.7 (3.3)	16.8 (6.4)	1.8 (2.6)

Participants excluded were significantly shorter, lighter, had lower lean mass, and were predominantly females (see supplementary information Table 1).

### Between-group differences

The unadjusted and adjusted effects of impact group on BMD, BMC, and area at total hip and femoral neck are presented in Table 2. Statistically significant differences in BMD and *Z*-scores at total hip and femoral neck between HIG and LIG compared to the CG were found, even after adjusting from age, sex, and BMI (*p* < 0.01). The HIG had significantly higher BMD than the LIG, with the CG recording lowest levels of BMD at total hip and femoral neck. Both the HIG and LIG had higher levels or BMC compared with the CG, whilst the HIG had significantly less mean femoral neck area than the CG in all three models (*p* < 0.05).

**Table 2:** Linear regression analysis with the effect of impact group on total hip and femoral neck

	Group	Crude			Model 1			Model 2		
		Coef.	95% conf.	*p*	Coef.	95% conf.	*p*	Coef.	95% conf.	*p*
Mean total hip BMD (g/cm^2^)	Low impact	0.124	0.077–0.171	< 0.001	0.077	0.029–0.126	0.002	0.066	0.020–0.112	0.005
	High impact	0.192	0.143–0.240	< 0.001	0.144	0.094–0.195	< 0.001	0.110	0.061–0.159	< 0.001
Mean total hip *Z*-score	Low impact	0.7	0.4–1.1	< 0.001	0.6	0.2–1.0	0.001	0.5	0.2–0.9	0.004
	High impact	1.2	0.9–1.6	< 0.001	1.1	0.7–1.5	< 0.001	0.8	0.4–1.2	< 0.001
Mean total hip BMC (g)	Low impact	7.7	5.1–10.4	< 0.001	5.4	3.2–7.7	< 0.001	4.8	2.7–6.9	< 0.001
	High impact	8.8	6.1–11.5	< 0.001	6.6	4.3–8.9	< 0.001	4.8	2.6–7.1	< 0.001
Mean total hip area (cm^2^)	Low impact	3.2	1.9–4.5	< 0.001	2.6	1.6–3.5	< 0.001	2.4	1.4–3.4	< 0.001
	High impact	2.0	0.6–3.3	< 0.001	1.4	0.4–2.5	0.006	1.0	− 0.1–2.0	0.066
Mean femoral neck BMD (g/cm^2^)	Low impact	0.142	0.091–0.192	< 0.001	0.097	0.044–0.149	< 0.001	0.085	0.036–0.135	0.001
	High impact	0.208	0.156–0.259	< 0.001	0.163	0.109–0.217	< 0.001	0.127	0.074–0.179	< 0.001
Mean femoral neck *Z*-score	Low impact	0.9	0.5–1.2	< 0.001	0.7	0.3–1.1	0.001	0.6	0.2–1.0	0.002
	High impact	1.3	1.0–1.7	< 0.001	1.2	0.8–1.6	< 0.001	0.9	0.5–1.3	< 0.001
Mean femoral neck BMC (g)	Low impact	0.8	0.2–1.4	0.011	0.3	− 0.3–0.9	0.388	0.2	− 0.4–0.8	0.529
	High impact	0.7	0.1–1.3	0.028	0.2	− 0.4–0.8	0.564	0.0	− 0.7–0.6	0.887
Mean femoral neck area (cm^2^)	Low impact	− 0.1	− 0.5–0.4	0.797	− 0.3	− 0.8–0.2	0.219	− 0.3	− 0.7–0.2	0.219
	High impact	− 0.5	− 0.9	0.042	− 0.7	− 1.2 to − 0.2	0.005	− 0.7	− 1.2 to − 0.2	0.006

Model 1—impact group + age + sex adjusted; Model 2—impact group + age + sex + BMI adjusted. Control group as the reference category

### Within-group differences

For all participants, irrespective of group, dominant–non-dominant hip BMD were highly correlated for all hip regions (*r*^2^ > 0.88) with the dominant side recording higher BMD. Bone density distribution for dominant and non-dominant hip per impact group are described in supplementary information Table 2. On average, BMD values were higher on the non-dominant side in the HIG and on the dominant side in the LIG and CG.

Hip asymmetries were not found between males or females. However, a significant group-by-side interaction on BMD was found (see Table 3 and supplementary information Figure 1).

**Table 3:** Linear regression analysis with the effect of side on total hip and femoral neck

	High-impact group			Low-impact group			Control group			*p* value for interaction		
										LIG vs. HIG	CG vs. LIG	CG vs. HIG
	Coef.	95% conf.	*p*	Coef.	95% conf.	*p*	Coef.	95% conf.	*p*			
Total hip BMD (g/cm^2^)	0.009	− 0.000–0.018	0.052	− 0.012	− 0.02 to − 0.004	0.004	− 0.006	− 0.017–0.005	0.294	0.001	0.429	0.048
Total hip *Z*-score	0.1	− 0.0–0.1	0.105	− 0.1	− 0.1 to − 0.0	0.008	− 0.1	− 0.1–0.0	0.169	0.002	0.685	0.041
Total hip BMC (g)	0.4	0.0–0.8	0.041	− 0.3	− 0.7–0.0	0.066	0.0	− 0.5–0.5	0.940	0.005	0.272	0.239
Total hip area (cm^2^)	0.1	− 0.1–0.2	0.402	0.1	− 0.1–0.2	0.435	0.2	− 0.1–0.4	0.160	0.966	0.498	0.531
Femoral neck BMD (g/cm^2^)	0.011	0.001–0.022	0.039	− 0.011	− 0.021 to − 0.000	0.040	− 0.155	− 0.031 to − 0.000	0.045	0.004	0.623	0.005
Femoral neck *Z*-score	0.1	− 0.0–0.2	0.082	− 0.1	− 0.2–0.0	0.057	− 0.1	− 0.2 to − 0.0	0.049	0.011	0.575	0.009
Femoral neck BMC (g)	0.1	− 0.1–0.2	0.385	0.0	− 0.2–0.1	0.485	0.0	− 0.2–0.2	0.864	0.294	0.589	0.750
Femoral neck area (cm^2^)	0.0	− 0.1–0.1	0.958	0.0	− 0.1–0.1	0.606	0.1	− 0.1–0.3	0.345	0.770	0.493	0.367

Dominant foot is the reference side

Table 3 shows the effect of side on bone mass at total hip and femoral neck stratified by impact group. BMD and BMC at the total hip were significantly higher (*p* ≤ 0.05) on the non-dominant side within the HIG, whereas the dominant hip had significantly higher BMD in the LIG (*p* < 0.05) at both ROIs. There were no significant differences in bone area between dominant and non-dominant sides at either the total hip or femoral neck for either impact group. The CG had higher femoral neck BMD and *Z*-score on the dominant side but no other differences were found. Significant interactions between CG and LIG with HIG were found. However, no evidence of difference in the effect of side with BMD was observed between LIG and CG (see Table 3 and supplementary information Figure 1).

**Fig. 1:**
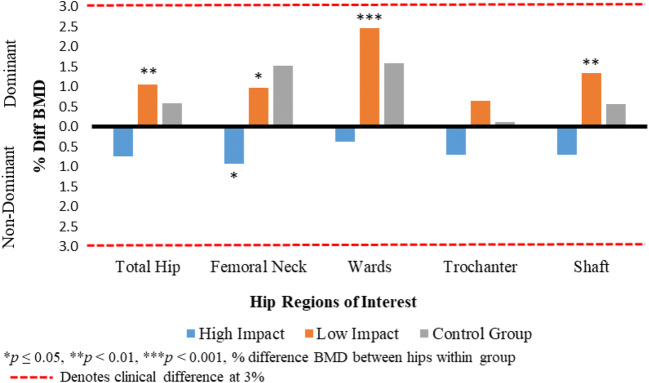
Percentage BMD difference between dominant and non-dominant feet for all hip ROIs

Percentage differences in BMD for all ROIs of the hip between the dominant and non-dominant sides are presented in Fig. 1. Statistically significant BMD differences were observed within groups in the total hip, femoral neck, wards, and shaft in the LIG with the dominant side having higher BMD values. This trend of higher BMD values on the dominant side was seen in the CG, albeit not significantly different at any ROIs. Within the HIG, BMD was higher on the non-dominant side with the femoral neck being significantly higher. However, differences between hips within all three groups were small and not clinically different (< 3%).

## Discussion

The results from this study suggest that participating in sport, regardless of ground impact severity, is significantly beneficial for BMD in the hip when compared to non-athletic controls, even after adjusting for age, sex, and BMI. Within impact groups, BMD values were on average higher in the non-dominant hip in the HIG, whereas BMD was higher in the dominant hip for the LIG and CG. Significant differences were observed in side-to-side comparison in the case groups; albeit these differences were relatively small and not clinically relevant (differences lower than 3%).

Within both impact groups, the BMD *Z*-scores were consistently higher than a score of zero for all ROIs, meaning that this young, healthy population had increased levels of BMD compared to the GE Healthcare’s reference population (see supplementary information Table 2). The CG *Z*-scores were consistently below the NHANES reference population. High-impact sports, including rugby, athletics, and powerlifting, presented the highest values of BMD at the hip and femoral neck. These findings are in line with previous studies [20–24]. Fredericson and colleagues [33] suggested that long-term exposure to high-impact activities such as running, jumping, accelerating, decelerating, and weight lifting is beneficial to bone density at all measured sites. Low-impact sports, such as swimming, cycling, and rowing, are predominantly non-weight-bearing activities resulting in lower ground force stresses on the body. The results from this study suggest that participating in low-impact sports is still beneficial for the development of bone in the hip when compared with controls. Competing in these sports may have a positive effect on bone not due to the ground impact forces but due to the repeated muscle contractions with repetitive, vigorous movements leading to increased mechanical loading and strain on the skeleton [29]. Interactions between HIG with LIG and CG were statistically significant, meaning that hip asymmetry is different depending on whether athletes are participating in high-impact sport or in low-impact sport/general population. The dominant side had a statistically significantly higher BMD within LIG at total hip and femoral neck, whereas the opposite trend was observed within the HIG. The non-dominant hip had significantly higher BMD across all ROIs, with the difference being significant (≤ 0.05) at total hip and femoral neck. One plausible explanation of this is that there could be frequent and increased loading of the non-dominant hip when completing the sporting task. Whilst the dominant leg is performing a given task (e.g. kicking a rugby ball), the non-dominant leg, which maintains the equilibrium in the upright position, exposes the loading effect of the body weight and exercise together and, therefore, increased BMD [34].

This research indicates that although there are statistically significant differences between dominant and non-dominant sides, these differences are relatively small and not clinically relevant. Therefore, these findings are in line with the current International Society for Clinical Densitometry’s 2015 guidelines [4] of scanning both the anteroposterior spine and either hip in all patients to assess bone health. Chilibeck and colleagues [32] suggested that a greater lifetime of preferential loading of the dominant limb resulted in a greater difference between limbs in an older population compared to a younger population. Therefore, future studies looking at the differences in BMD between sides should focus on dominant limb use in older/more-experienced athletes with a longer lifetime exposure to sport-specific loading patterns.

## Strengths and limitations

The strengths of this study are that, to the authors’ knowledge, this is one of the largest cross-sectional studies investigating BMD differences between dominant and non-dominant hips with respect to impact loading in a young sporting population. All participants were recruited from the same university with a similar age, socioeconomic background, with the case groups competing at the same university standard of play. Hip dominance was determined by the preference of foot, assessed via a self-reported measure, rather than preference of hand. This was assessed and reported due to the high use and load placed on the feet by sport, which it is inferred to have a stronger relationship to the hip than the dominant hand. Therefore, participants who did not specify a dominant foot or declared both feet dominant were excluded to remove potential ambiguous data. Other strengths are that data consistency across recordings was ensured by having the DXA scans being performed and analysis by the same radiographer, using the same software and reference population, in a scanner that demonstrated high levels of precision (0.25–0.27% error margin) throughout the duration of the entire study.

As with any cross-sectional study, the cause and effect relationship between impact and bone density cannot be determined, and that any confounding variables cannot be ruled out as an explanation for these findings. However, as each participant acts as their own control, due to the measurement of dominant and non-dominant hips within a person, confounding should act equally for all ROIs for this analysis. The results from this young, high-level university sporting population may not be extrapolated to the lower level sporting populations or general non-sporting and older populations. In addition, a detailed training log or sporting history was not assessed, so some additional information about differences in exposure to previous sporting/physical activity and the impact it has on BMD may have been missed. Finally, the lack of significant associations may be due to type II errors; therefore, future studies with an increased sample size are suggested.

## Conclusion

Physical activity and sport play an important role in the positive development of BMD in young athletes. Regardless of ground force impact severity, people who participate in sport had increased levels of hip BMD when compared to the non-sporting controls and the NHANES reference population. There were statistical, but no clinical differences between dominant and non-dominant hip BMDs supporting the ISCD’s guidelines of scanning either hip to assess bone health, along with the anteroposterior spine. Future studies are required to assess the long-term effects of a variety of sports on BMD longitudinally, specifically reporting the associations of previous sporting/physical activity levels with hip and total body BMD.

## Electronic supplementary material

ESM 1 (DOCX 12 kb)

ESM 2 (DOCX 16 kb)

ESM 3 (DOCX 66 kb)
