# Supplementary material for: Dominant vs. non-dominant hip comparison in bone mineral density in young sporting athletes
Source: Arch Osteoporos. 2019 May 25;14(1):54. doi: 10.1007/s11657-019-0605-2 (PMC6535155; doi:10.1007/s11657-019-0605-2)
Supplement: Supplementary file 1 — (DOCX 12 kb) [file 11657_2019_605_MOESM1_ESM.docx]

**Supplementary Table 1.** Baseline differences between included vs excluded participants

|  | Included | Excluded | *p* |
| --- | --- | --- | --- |
| n | 242 | 59 | - |
| Male : Female ratio | 55:45 | 29:71 | - |
| Case : control ratio | 80:20 | 76:24 | - |
| Age (years) | 24.6 (4.9) | 23.5 (4.0) | 0.106 |
| Height (cm) | 176.0 (9.9) | 173.0 (9.2) | **0.032** |
| Weight (kg) | 72.6 (12.8) | 67.9 (11.7) | **0.010** |
| BMI (kg/m^2^) | 23.3 (2.8) | 22.5 (2.5) | 0.058 |
| Lean Mass (kg) | 55.0 (12.8) | 50.0 (10.9) | **0.006** |
| Total Fat Mass (%) | 21.4 (8.6) | 23.3 (8.3) | 0.125 |
| Physical activity & Sport (Hours/week) | 10.9 (7.5) | 11.7 (7.9) | 0.450 |
|  |  |  |  |
| Values are mean (SD) |  |  |  |
