# Supplementary material for: Dominant vs. non-dominant hip comparison in bone mineral density in young sporting athletes
Source: Arch Osteoporos. 2019 May 25;14(1):54. doi: 10.1007/s11657-019-0605-2 (PMC6535155; doi:10.1007/s11657-019-0605-2)
Supplement: Supplementary file 2 — (DOCX 16 kb) [file 11657_2019_605_MOESM2_ESM.docx]

|  | | High Impact (n = 89) | | Low Impact (n = 105) | | Control Group (n = 48) | |
| --- | --- | --- | --- | --- | --- | --- | --- |
|  |  | Dominant | Non-Dominant | Dominant | Non-Dominant | Dominant | Non-Dominant |
| Total Hip | BMD (g/cm^2^) | 1.213 (0.154) | 1.222 (0.154) | 1.156 (0.136) | 1.144 (0.135) | 1.029 (0.107) | 1.023 (0.117) |
|  | Z-score* | 1.1 (1.0) | 1.1 (1.0) | 0.7 (1.0) | 0.6 (1.0) | -0.1 (0.8) | -0.1 (0.9) |
|  | BMC (g) | 42.5 (8.4) | 42.9 (8.5) | 41.8 (7.5) | 41.5 (7.4) | 33.9 (6.4) | 33.9 (6.9) |
|  | Area (cm^2^) | 34.8 (3.7) | 34.9 (3.7) | 36.1 (4.0) | 36.1 (4.0) | 32.8 (4.0) | 33.0 (4.1) |
| Femoral Neck | BMD (g/cm^2^) | 1.194 (0.164) | 1.205 (0.178) | 1.139 (0.144) | 1.128 (0.140) | 1.000 (0.111) | 0.985 (0.128) |
|  | Z-score* | 0.9 (1.2) | 1.0 (1.3) | 0.5 (1.1) | 0.5 (1.0) | -0.3 (0.8) | -0.4 (1.0) |
|  | BMC (g) | 6.8 (1.7) | 6.9 (1.6) | 6.9 (1.8) | 6.9 (1.8) | 6.1 (1.8) | 6.2 (2.0) |
|  | Area (cm^2^) | 5.7 (1.2) | 5.7 (1.1) | 6.1 (1.4) | 6.1 (1.4) | 6.1 (1.5) | 6.2 (1.5) |
| Wards | BMD (g/cm^2^) | 1.089 (0.181) | 1.093 (0.183) | 1.035 (0.164) | 1.010 (0.161) | 0.895 (0.135) | 0.881 (0.139) |
|  | Z-score* | 0.9 (1.3) | 0.9 (1.3) | 0.5 (1.2) | 0.3 (1.2) | -0.3 (1.0) | -0.4 (1.0) |
|  | BMC (g) | 3.4 (1.0) | 3.4 (1.0) | 3.3 (0.8) | 3.2 (0.8) | 2.5 (0.7) | 2.5 (0.8) |
|  | Area (cm^2^) | 3.1 (0.6) | 3.1 (0.6) | 3.2 (0.7) | 3.2 (0.6) | 2.8 (0.6) | 2.8 (0.6) |
| Trochanter | BMD (g/cm^2^) | 0.990 (0.155) | 0.997 (0.154) | 0.942 (0.127) | 0.936 (0.126) | 0.809 (0.103) | 0.808 (0.107) |
|  | Z-score* | 0.8 (1.2) | 0.9 (1.2) | 0.4 (1.1) | 0.3 (1.1) | -0.6 (0.9) | -0.6 (1.0) |
|  | BMC (g) | 14.0 (4.0) | 14.1 (4.1) | 13.7 (3.6) | 13.6 (3.5) | 10.1 (2.5) | 10.1 (2.6) |
|  | Area (cm^2^) | 13.9 (2.5) | 14.0 (2.5) | 14.4 (2.5) | 14.4 (2.4) | 12.4 (2.0) | 12.4 (2.1) |
| Shaft | BMD (g/cm^2^) | 1.425 (0.175) | 1.435 (0.175) | 1.362 (0.168) | 1.344 (0.164) | 1.232 (0.135) | 1.225 (0.146) |
|  | BMC (g) | 21.7 (3.5) | 21.9 (3.5) | 21.1 (3.0) | 21.0 (3.0) | 17.6 (2.5) | 17.6 (2.7) |
|  | Area (cm^2^) | 15.2 (1.2) | 15.2 (1.3) | 15.5 (1.2) | 15.6 (1.3) | 14.3 (1.2) | 14.4 (1.2) |
| Values are mean (SD)  * 3 High Impact, 1 Low Impact & 1 Control did not have a z-score for being < 20 years old | | | | | | |  |

**Supplementary Table 2.** Distribution of bone mass results per impact group stratified by dominance
