# Supplementary material for: Dominant vs. non-dominant hip comparison in bone mineral density in young sporting athletes
Source: Arch Osteoporos. 2019 May 25;14(1):54. doi: 10.1007/s11657-019-0605-2 (PMC6535155; doi:10.1007/s11657-019-0605-2)

**Supplementary Figure 1.** Side by Impact Group interactions for Total Hip and Femoral Neck BMD adjusted for Sex & BMI


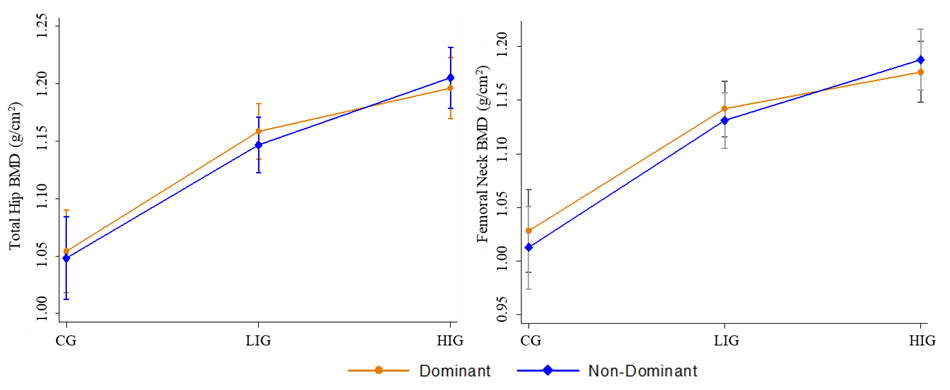

Supplement: Supplementary file 3 — (DOCX 66 kb) [file 11657_2019_605_MOESM3_ESM.docx]
